# Draft Genome Sequences of Three Clinical Isolates of *Tannerella forsythia* Isolated from Subgingival Plaque from Periodontitis Patients in the United States

**DOI:** 10.1128/genomeA.01286-16

**Published:** 2016-12-01

**Authors:** Graham P. Stafford, Roy R. Chaudhuri, Violet Haraszthy, Valentin Friedrich, Christina Schäffer, Angela Ruscitto, Kiyonobu Honma, Ashu Sharma

**Affiliations:** a Integrated Biosciences Group, School of Clinical Dentistry, University of Sheffield, Sheffield, United Kingdom; b Department of Molecular Biology and Biotechnology, University of Sheffield, Sheffield, United Kingdom; c Department of Oral Biology, University at Buffalo, SUNY, Buffalo, New York, USA; d Department of NanoBiotechnology, NanoGlycobiology Unit, Universität für Bodenkultur Wien, Muthgasse, Vienna, Austria

## Abstract

We report the genome sequences of three clinical isolates of *Tannerella forsythia* from the subgingival plaque of periodontitis patients attending clinics at the School of Dental Medicine, University at Buffalo. The availability of these genome sequences will aid the understanding of the pathogenesis of periodontitis.

## GENOME ANNOUNCEMENT

Periodontitis is a common condition in humans, estimated to affect approximately 20% of those 35 to 44 years old globally (1) and 47% of the U.S. population (2). Periodontitis describes a group of conditions characterized by chronic inflammation of the gingiva, recession of gums, degradation of the supporting structures of the tooth, including the periodontal ligament and alveolar bone, and formation of periodontal pockets that act as a niche for colonizing bacteria (3). It is increasingly linked with systemic sequelae such as rheumatoid arthritis (4, 5), diabetes (6), and cardiovascular disease (7) and represents a major global health burden.

Unlike other conditions, periodontitis is considered a polymicrobial disease with the presence of certain organisms representative of a generalized dysbiosis contributing to the observed symptoms (8). This smaller group of periodontal pathogens is often termed the “red complex” after the pioneering work of Socransky and colleagues (9). Within this red complex of bacteria, *Tannerella forsythia* has long been an understudied organism, mainly due to difficulties in cultivation and isolation owing to fastidious growth requirements, such as requirement for *N*-acetylmuramic acid (MurNAc) (10).

We report here the draft genome sequences of three novel *T. forsythia* strains, isolated from the subgingival plaque of patients attending the clinic at the School of Dental Medicine, University at Buffalo, in 2013. Briefly, plaque samples from periodontal pockets >5 mm were serially diluted and plated on tryptic soy blood agar plus MurNAc plates (11) and incubated anaerobically (37°C), before sialidase-positive colonies were picked and grown in broth cultures and gDNA was isolated. Colonies with a 16S rDNA sequence identifying them as strains of *T. forsythia* via comparison to ATCC 43037 were subsequently passaged and maintained on agar. Three strains were isolated and named UB4, UB20, and UB22.

DNA was extracted using Promega Wizard kits before sequencing on the Illumina platform using 2 × 300-bp paired-end sequencing either commercially at MrDNA, Shallowater, TX, USA (UB22), or via MicrobesNG, United Kingdom (UB4 and UB20). Contigs were assembled and scaffolded using SPAdes version 3.8.0 with the options “-careful, -cov_cutoff 10,” followed by automated annotation using Prokka (12). The scaffolds were reordered and reoriented based on NUCmer (13) comparisons with the published *T. forsythia* 92A2 genome sequence (CP003191), and the annotations were manually inspected and refined using ACT (14).

General properties of the genomes are summarized in Table 1. The genomes are generally well conserved with >90% similarity for most genes and very similar GC contents. Recent work has revealed several virulence determinants in this organism (8, 15, 16). First, a TLR-2 activating surface protein called BspA present in all three strains is well conserved in the C-terminal region. Furthermore, the sialic acid utilization *nan* gene cluster can be found in all strains (17), as well as genes encoding the S-layer (*tfsAB*) and a novel MurNAc uptake system (18).

**TABLE 1:** Metadata for *T. forsythia* strains isolated from subgingival plaque from patients in Buffalo, NY, USA

Strain name	Assembly accession no.	Run accession no.	Source tissue	Total assembly size (bp)	*N*_50_	G+C content (%)	No. of scaffolds
UB4	FMMN01000000	ERR1633772	Subgingival plaque	3,232,117	136,594	47.20	71
UB20	FMMM01000000	ERR1633773	Subgingival plaque	3,252,817	140,079	47.05	92
UB22	FMML01000000	ERR1633774	Subgingival plaque	3,272,088	93,893	47.07	97

The availability of these draft genome sequences will improve understanding of *T. forsythia* and aid in forging new treatments for globally important periodontitis disease.

### Accession number(s).

The accession numbers for the draft sequences of the three *T. forsythia* strains and scaffold sequences deposited in the European Nucleotide Archive (ENA) and NCBI databases are listed in Table 1.
